# mSphere of Influence: The age of antifungal resistance

**DOI:** 10.1128/msphere.00009-26

**Published:** 2026-08-20

**Authors:** Norman van Rhijn

**Affiliations:** 1 Department of Biology, University of Oxford 6396, Oxford, United Kingdom; Virginia-Maryland College of Veterinary Medicine, Blacksburg, Virginia, USA

**Keywords:** antifungal resistance, fungal infections, mycology, climate change, emerging pathogens

## Abstract

Norman van Rhijn works in the field of One Health relating to fungal pathogens and antifungal resistance studying the factors that shape the emergence and spread of fungal diseases. In this mSphere of Influence article, they reflect on how the work by M. E. Gorris, K. K. Treseder, C. S. Zender, and J. T. Randerson (GeoHealth 3:308–327, 2019, https://doi.org/10.1029/2019GH0002) made an impact on them by evidencing that environmental change directly impacted the change in habitat of *Coccidioides immitis*, the fungal pathogen responsible for Valley fever. This work has shaped their approach toward understanding fungal pathogens and environmental change driving antifungal resistance.

## COMMENTARY

Throughout my academic career, I have been driven to understand how fungal pathogens interact with the environments they inhabit and how changes in those environments influence human disease. I became particularly interested in the idea that climate change reshapes the natural homes of pathogenic fungi. This perspective fundamentally changed how I thought about fungal disease: not only as a clinical problem occurring within individual patients but also as an ecological and evolutionary problem. It led me to view the emergence of fungal pathogens and antifungal resistance as interconnected consequences of environmental change and fungal adaptation. One of the first examples of this was the expansion of Valley fever/coccidioidomycosis in relation to the climate changing (1). *Coccidioides immitis* is geography-bound to thrive in areas with low rainfall and high temperature, more specifically the southwest of the United States. With a changing climate, this fungal pathogen is moving northward and eastward, spanning a larger endemic area, resulting in more human exposure. Similarly, in Europe, *Cryptococcus gattii* VGI is originally bound to areas around the Mediterranean. However, it has now been isolated from areas all the way up into north Europe (2). Expansion of geography is also predicted for *Aspergillus niger* species complex and *Aspergillus flavus* species complex, and increasing number of infections have already been observed in California associated with high levels of azole resistance (3, 4). The spectrum of species causing candidiasis and candidemia are also changing as more infections are associated with non-*albicans Candida* (NAC) species (5). NAC species are more commonly associated with resistance to the azole, fluconazole, and therefore require species-level diagnosis.

Resistance in *Aspergillus fumigatus* is one of the clearest examples of environmental change driving antifungal resistance. More azole antifungals are used every year to combat fungal plant pathogens (6). While *A. fumigatus* is not the primary target of agriculturally used azoles, these organisms are exposed to it, driving selection for azole resistance. Mutations in the gene and promoter of the target of the azoles, *erg11/cyp51a*, mostly commonly TR34/L98H and TR46**/**Y121F/T289A, have become a genetic signature of the environmental route of resistance acquisition (7). Population-genomic analyses have identified closely related azole-resistant *A. fumigatus* isolates in clinical and environmental samples, supporting environmental acquisition of resistant infections (8). This is further evidenced by the fact that *A. fumigatus* isolates that are azole-resistant are also commonly resistant to fungicides, which are solely used in agriculture (e.g., methyl benzimidazole carbamate and quinone outside inhibitor fungicides) (9). Recent findings have also indicated that some multi-azole-resistant populations of *A. fumigatus* possess elevated mutation rates, potentially increasing their ability to evolve resistance to novel fungicides and antifungals (10).

The most prominent example of an NAC that has emerged over the last 10 years is *Candidozyma* (*Candida*) *auris*. Since its initial recognition in 2009, genetically distinct lineages have appeared across the whole world (11). Early whole-genome and epidemiological investigations showed that many isolates were resistant to fluconazole, some were resistant to amphotericin B or echinocandins, and a proportion displayed resistance to more than one major antifungal class. It has been hypothesized that this is the first fungal pathogen that has evolved due to climate change and that many of its traits are the result of its environmental niche (12). *C. auris* is not the only novel fungal pathogen that has emerged in the last two decades. *Trichophyton indotineae* is a dermatophyte associated with extensive and antifungal-resistant skin and nail infections (13). The emergence of this particular lineage of *Trichophyton* has yet to be further investigated. However, this highlights that antifungal resistance is seemingly a trait that is selected for and becoming the norm rather than the exception, demanding that the age of antifungal resistance be treated as a central component of antimicrobial resistance policy rather than as a specialized or secondary concern.

## References

[1] Gorris ME, Treseder KK, Zender CS, Randerson JT. 2019. Expansion of coccidioidomycosis endemic regions in the United States in response to climate change. GeoHealth 3:308–327. doi:10.1029/2019GH000209 PMC7007157 32159021

[2] Chowdhary A, Randhawa HS, Boekhout T, Hagen F, Klaassen CH, Meis JF. 2012. Temperate climate niche for *Cryptococcus gattii* in northern Europe. Emerg Infect Dis 18:172–174. doi:10.3201/eid1801.111190 PMC3310122 22261398

[3] Uzzell C, Shelton J, van Rhijn N. 2026. Climate change-driven geographical shifts in *Aspergillus* species and the implications for plant and human health. iScience 29:114911. doi:10.1016/j.isci.2026.114911 PMC12933601 41756734

[4] Wang Y, Aziz M, Bush K, Davis GS, Foster JT, Hallstrøm S, Jones A, Keim PS, Leventis R, Liu CM, Nelson SG, Park DE, Pomichowski M, Quinlivan V, Rayens E, Sahl JW, Sass J, Skela J, Sung E, Villani J, Zimmerman M, Stegger M, Tartof SY, Price LB. 2025. Triazole resistance and misidentification of *Aspergillus tubingensis* in Southern California. JAMA Netw Open 8:e2543630. doi:10.1001/jamanetworkopen.2025.43630 PMC12679326 41343218

[5] Whaley SG, Berkow EL, Rybak JM, Nishimoto AT, Barker KS, Rogers PD. 2017. Azole antifungal resistance in *Candida albicans* and emerging non-*albicans Candida* species. Front Microbiol 7:2173. doi:10.3389/fmicb.2016.02173 PMC5226953 28127295

[6] van Rhijn N, Rhodes J. 2025. Evolution of antifungal resistance in the environment. Nat Microbiol 10:1804–1815. doi:10.1038/s41564-025-02055-y 40730910

[7] Snelders E, Camps SMT, Karawajczyk A, Schaftenaar G, Kema GHJ, van der Lee HA, Klaassen CH, Melchers WJG, Verweij PE. 2012. Triazole fungicides can induce cross-resistance to medical triazoles in *Aspergillus fumigatus*. PLoS One 7:e31801. doi:10.1371/journal.pone.0031801 PMC3291550 22396740

[8] Rhodes J, Abdolrasouli A, Dunne K, Sewell TR, Zhang Y, Ballard E, Brackin AP, van Rhijn N, Chown H, Tsitsopoulou A, et al.. 2022. Population genomics confirms acquisition of drug-resistant *Aspergillus fumigatus* infection by humans from the environment. Nat Microbiol 7:663–674. doi:10.1038/s41564-022-01091-2 PMC9064804 35469019

[9] Celia-Sanchez BN, Mangum B, Gómez Londoño LF, Wang C, Shuman B, Brewer MT, Momany M. 2024. Pan-azole- and multi-fungicide-resistant *Aspergillus fumigatus* is widespread in the United States. Appl Environ Microbiol 90:e01782-23. doi:10.1128/aem.01782-23 PMC11022549 38557086

[10] Bottery MJ, van Rhijn N, Chown H, Rhodes JL, Celia-Sanchez BN, Brewer MT, Momany M, Fisher MC, Knight CG, Bromley MJ. 2024. Elevated mutation rates in multi-azole resistant *Aspergillus fumigatus* drive rapid evolution of antifungal resistance. Nat Commun 15:10654. doi:10.1038/s41467-024-54568-5 PMC11649685 39681549

[11] Lockhart SR, Etienne KA, Vallabhaneni S, Farooqi J, Chowdhary A, Govender NP, Colombo AL, Calvo B, Cuomo CA, Desjardins CA, Berkow EL, Castanheira M, Magobo RE, Jabeen K, Asghar RJ, Meis JF, Jackson B, Chiller T, Litvintseva AP. 2017. Simultaneous emergence of multidrug-resistant *Candida auris* on 3 continents confirmed by whole-genome sequencing and epidemiological analyses. Clin Infect Dis 64:134–140. doi:10.1093/cid/ciw691 PMC5215215 27988485

[12] Casadevall A, Kontoyiannis DP, Robert V. 2019. On the emergence of *Candida auris*: climate change, azoles, swamps, and birds. mBio 10:e01397-19. doi:10.1128/mBio.01397-19 PMC6650554 31337723

[13] Rhodes J, Hui ST, Dellière S, Summerbell RC, Scott JA, Kaur A, Barton RC, Leitao R, Hemmings S, Goiriz R, Lambourne J, Farrer RA, Schelenz S, Hay RJ, Borman AM, Chowdhary A, Abdolrasouli A, Fisher MC. 2026. Emerging terbinafine-resistant *Trichophyton indotineae* between 2018 and 2023: a multinational genomic epidemiology study. Lancet Microbe 7:101273. doi:10.1016/j.lanmic.2025.101273 41581521

